# Key Factors that Promote Low-Value Care: Views of Experts From the United States, Canada, and the Netherlands

**DOI:** 10.34172/ijhpm.2021.53

**Published:** 2021-06-19

**Authors:** Eva W. Verkerk, Simone A. Van Dulmen, Karen Born, Reshma Gupta, Gert P. Westert, Rudolf B. Kool

**Affiliations:** ^1^Department of IQ Healthcare, Radboud Institute for Health Sciences, Radboud University Medical Center, Nijmegen, The Netherlands.; ^2^Institute for Health Policy, Management & Evaluation, University of Toronto, Toronto, ON, Canada.; ^3^University of California Health, Sacramento, CA, USA.

**Keywords:** Low-Value Care, De-Implementation, Medical Overuse, Overtreatment, Overdiagnosis, Disinvestment

## Abstract

**Background:** Around the world, policies and interventions are used to encourage clinicians to reduce low-value care. In order to facilitate this, we need a better understanding of the factors that lead to low-value care. We aimed to identify the key factors affecting low-value care on a national level. In addition, we highlight differences and similarities in three countries.

**Methods:** We performed 18 semi-structured interviews with experts on low-value care from three countries that are actively reducing low-value care: the United States, Canada, and the Netherlands. We interviewed 5 experts from Canada, 6 from the United States, and 7 from the Netherlands. Eight were organizational leaders or policy-makers, 6 as low-value care researchers or project leaders, and 4 were both. The transcribed interviews were analyzed using inductive thematic analysis.

**Results:** The key factors that promote low-value care are the payment system, the pharmaceutical and medical device industry, fear of malpractice litigation, biased evidence and knowledge, medical education, and a ‘more is better’ culture. These factors are seen as the most important in the United States, Canada and the Netherlands, although there are several differences between these countries in their payment structure, and industry and malpractice policy.

**Conclusion:** Policy-makers and researchers that aim to reduce low-value care have experienced that clinicians face a mix of interdependent factors regarding the healthcare system and culture that lead them to provide low-value care. Better awareness and understanding of these factors can help policy-makers to facilitate clinicians and medical centers to deliver high-value care.

## Background

**Key Messages**

**Implications for policy makers**

- Policy-makers and researchers that aim to reduce low-value care have experienced that clinicians act in a system and culture that promotes low-value care.
- The delivery of high-value care can be supported with policy changes regarding the payment system, the influence of the pharmaceutical and medical device industry, and medical malpractice policy.
- Increased awareness of the bias in medical knowledge and the ‘more is better’ culture can help policy-makers to better support clinicians and medical centers to deliver high-value care to their patients.

**Implications for the public**

Many patients receive care that does not benefit them, but it does cause harm and wastes limited resources. Reducing this so-called low-value care will improve the quality and safety of care and the sustainability of our healthcare systems. However, this is not easy. Clinicians and patients act in a system and culture that promotes low-value care. We found that the payment system, the pharmaceutical and medical device industry, fear of malpractice litigation, biased evidence and knowledge, medical education, and a ‘more is better’ culture promoted low-value care. Changing this system can support clinicians and medical centers to provide only high-value care to their patients.

Low-value care is a global problem that places a strain on healthcare systems.^1^ Low-value care harms patients and stresses the limited healthcare resources. In the United States, an estimated 75.7 to 101.2 billion dollars were spent in 2019 on overtreatment or low-value care.^2^ Reducing low-value care is therefore a necessary step towards reaching the triple aim of healthcare: improving healthcare and population health while reducing costs.^3^

 In many countries, the number of national and local initiatives targeting low-value care is rising.^1^ The largest of them is the Choosing Wisely^®^ campaign, which has been adopted by over 20 countries since its launch.^4^ The United States first initiated the campaign in April 2012, followed by the Netherlands in November 2012 and Canada in April 2014. Other key initiatives have developed by Costs of Care Inc, the Lown Institute, and the High-value Practice Academic Alliance.^5-7^ Several initiatives show success in reducing low-value care.^8-11^ Others show less success; they sometimes cannot or can only temporarily overcome the factors that lead to the problem.^12-14^ Therefore, experts suggest changing systems rather than trying to change clinician behavior to create greater reductions in low-value care delivery.^15^

 In order to create a system that facilitates the delivery of high-value care, it is vital to understand what factors lead to low-value care and through what mechanisms.^14^ There have been multiple studies that identify factors experienced by clinicians, or factors that lead to a specific low-value care practice. However, few studies focus on national-level factors that promote the delivery of many types of low-value care. Saini et al described factors leading to overuse and underuse on the global, national, regional and local level including available resources, social and political contract, the state of scientific knowledge, the configuration and capacity of the delivery system, and financing mechanisms.^16^ The authors suggested that achieving high-value care requires an understanding of and attentiveness to all these dimensions.^16^ Pathirana et al found in literature that culture, the health system, industry and technology, professionals’ knowledge and fears, and patients’ expectations can lead to low-value care.^17^ These studies describe many factors that limit high-value care, and an assessment of the key factors can help policy-makers prioritize their improvement efforts in daily practice. Since 2012, the Choosing Wisely campaigns have worked on reducing low-value care, and their experiences and knowledge can provide insight into this complex problem.

 In order to support countries in prioritizing their actions aimed at reducing low-value care, we aimed to identify and deepen the knowledge on the key factors affecting low-value care by interviewing experts from three leading Choosing Wisely countries: the United States, Canada, and the Netherlands. In addition, we highlight differences and similarities in these three countries.

## Methods

 We performed semi-structured interviews with experts on low-value care from three countries: the United States, Canada, and the Netherlands. These three countries have increased awareness of low-value care, engaged societies and clinicians, improved medical education and stimulated quality improvement efforts.^14,18,19^ Each country has a different healthcare system. The United States has a mix of public and private financing, while Canada and the Netherlands have predominantly publicly financed health systems. In the United States and Canada the central federal government takes part in guiding national trends in healthcare delivery though programs as Medicare and Medicaid, while a large part of the healthcare policy is made by the provinces and territories or states. In the Netherlands, the central government manages primary and secondary care policy.

###  Participants

 We selected from our professional networks a convenience sample of 20 policy-makers and researchers with experience in identifying and reducing low-value care, distributed over the three countries. This was defined as having led at least one initiative to reduce low-value care, having evaluated such initiatives, or being responsible for reducing low-value care in an organization. We used purposive sampling to include experts from different institutes and programs and with different experiences. For example, we selected experts involved in the Choosing Wisely campaigns, researchers that focus on low-value care, and leaders of various organizations that aim to reduce low-value care. All experts were invited to participate and received information about the interviews by email. Eighteen of 20 experts gave oral consent to participate. More information regarding our methods can be found in the reporting guideline in Supplementary file 1.

###  Interview Guide

 The interviews started with an open-ended question on what factors promote low-value care practices according to the expert’s experiences. The factors that emerged were further explored with follow-up questions. Next, they were asked about a list of factors that influence low-value care in order to remind the expert of potential factors. From Saini et al,^16^ we selected national and global level factors of low-value care. We added factors thought to be relevant from the determinants of practice of Flottorp et al.^20^ Lastly, we asked experts what they believed to be the most important factors. All authors discussed this interview guide until they reached consensus. The interviewer tested the guide by interviewing a project manager from Choosing Wisely Canada. We added additional factors that emerged during the interviews in subsequent interviews. The final interview guide can be found in Supplementary file 2.

###  Data Collection

 We conducted face-to-face interviews with five Dutch experts and three Canadian experts and ten telephone interviews. One author (EWV) performed and audio-recorded all the interviews from August 2017 to December 2017. No new information emerged from the last two interviews and saturation was reached.

###  Analysis

 We used the qualitative data analysis software Atlas.ti 8.0.34 to analyze the transcribed interviews using inductive thematic analysis. In this approach, the analysis is data-driven to guide researchers to create overarching themes without a pre-existing frame.^21^ The analysis started by giving initial codes to relevant quotes. EWV and SAvD independently coded three interviews and discussed their coding until they reached consensus. EWV coded subsequent interviews and discussed her analysis regularly with SAvD. Subsequently, they grouped codes into categories derived from the data through continuous comparison and review. Based on the data, EWV and SAvD first selected the most important factors. All authors discussed the categories and selection of key factors through several rounds of discussion. The authors only included factors that promote low-value care on a national or global level. This was defined as factors that are related to national policy or that promote the delivery of many types of low-value care. Factors that were related to local policy, that promote the delivery of a specific low-value care practice, or act on a micro level were excluded. Examples of excluded factors are ‘lack of shared decision-making,’ ‘absence of sharing medical records or test results between providers,’ and ‘clinical uncertainty in predicting value of care for individual patient.’ National-level factors that were reported but excluded because they were not seen as key by most participants were for example ‘insufficient primary care,’ ‘performance measures that reward overuse,’ and ‘lack of cost sharing by patients, such as copayments or deductibles.’

## Results

 Of the 18 experts, 5 (28%) were from Canada, 6 (33%) from the United States, and 7 (39%) from the Netherlands. Eleven (61%) experts had a background as a clinician. Eight (44%) were characterized as organizational leaders or policy-makers, 6 (33%) as low-value care researchers or project leaders, and 4 (22%) were both. Twelve (67%) experts were male, and 1 Dutch expert had studied low-value care in the United States. Supplementary file 3 shows the characteristics of each expert. The analysis resulted in seven factors that promote low-value care, categorized into three themes (Figure). Table 1 shows sample quotes per factor.

**Figure:**
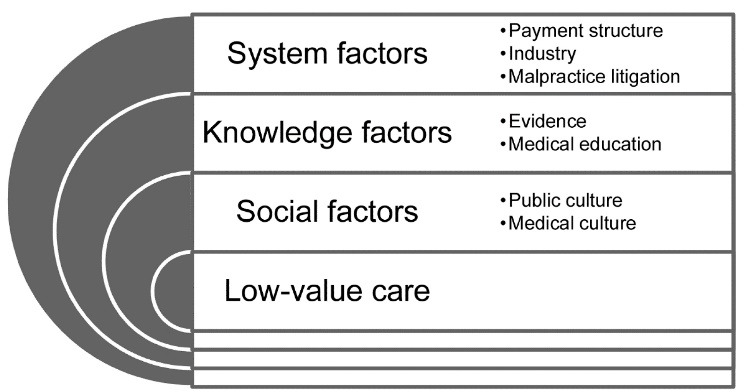
Seven Key Factors that Promote Low-Value Care.

**Table 1:** Sample Quotes From Experts

**Category**	**Factor **	**Quote**
System factors	Payment structure	*“Somebody gets paid for doing that care, and if you’re reducing it you’re affecting people’s income. And they can mount very powerful campaigns against you”* (Expert number 8 from Canada). *“There are certainly people who are ordering unnecessary tests because simply it increases their payments from their salary. (…) It's not like there is any downside to you as a physician in fact there is only upside. So I think the bigger issue of fee for service in the system that we have is that it just provide, it doesn't provide any counterbalance to the drive to get more testing. And so having you know value based payments or other models that at least provide some external counterbalance to it” *(Expert number 4 from the United States).
	Industry	*“They can push in a lot of ways, through patient organizations, for example. (..) they will talk to the press, to politicians, and to increase pressure they will give stories from patients that show how terrible their disease is”* (Expert number 16 from the Netherlands). *“They’ll give money to the Canadian diabetes association. And they’ll argue for tighter hemoglobin a1c control. Let’s not use 8,5 in the elderly, but use 7,5 in the elderly. Well what does that mean? We will have to treat more people and use drugs”* (Expert number 11 from Canada).
	Malpractice litigation	*“The risk of a doctor walking into an office tomorrow and doing something to a patient that causes a lawsuit is very low. But the potential consequence of that scares the crap out of most doctors which is why the practice the way they do it” *(Expert number 11 from Canada). “*A frequent complaint is that a patient was diagnosed too late or incorrectly. (…) Giving each patient that has a cough an X-ray to detect lung cancer might prevent you from missing that one patient, but you might make 100 unnecessary X-rays in the process. As a clinician you are almost never sued for what you have done, but more often for what you did not do or did too late” *(Expert number 17 from the Netherlands).
Knowledge factors	Evidence	*“I think it's a huge problem because almost all major clinical trials at the ultimate stage before approvals are funded by commercial sponsors and commercial entities” *(Expert number 4 from the United States). *“A hospital reports that they operate less on inguinal hernias since they have better conversations with patients, I believe about 10%. (…) But we need to study the effects thoroughly before applying this more widely. (…) We might find out in a few years that these people end up with much bigger problems” *(Expert number 17 from the Netherlands).
	Medical education	*“…you wanna be thorough and show the attending physician that you thought of all these diseases and you ruled them out. (…) It’s just baked in like, you get rewarded for being thorough and thinking maybe this is rare disease…*” (Expert number 10 from Canada). *“I think what drives a lot is the training. That insists upon no a stone unturned. Nothing, you know, you wanna nail down the diagnosis. You need as many tests as you can”* (Expert number 1 from the United States).
Social factors	Public culture	*“There is a tendency to help. Clinicians want to mean something to a patient, and the patient does not want to leave without a prescription with the idea that at least something has been done”* (Expert number 18 from the Netherlands). *“I would say it’s the lack of health literacy around the benefits and harms of a treatment. People don’t know. I mean people, everyone. Patients, doctors, hospitals administrators, politicians, health officials, they have an overblown sense of the benefit and an underappreciative sense of the harms when it comes to a lot of treatments” *(Expert number 8 from Canada).
	Medical culture	*“I think in the end most doctors just want to do the right thing for their patients right and they don't want the patients to have a bad outcome”* (Expert number 5 from the United States). “*We don’t always evaluate the efficacy and sometimes people assume that all innovation is good innovation. People assume that anything new has to be good and that is unfortunately not the case” *(Expert number 7 from Canada).

###  System Factors

####  Payment Structure

 According to most experts, payment structure emphasizing volume over value impacts the uptake of de-implementing low-value care initiatives. The experts described that fee-for-service payment models are a barrier to reducing this low-value care as clinicians have concerns about their ability to sustain revenue. With clinicians incentivized to do and bill for more, some focus efforts on protecting the viability of their jobs and their specialty. Some, however, even in light of these barriers advocate for the reduction of low-value care. For example, a Choosing Wisely recommendation from the Netherlands aims to reduce unnecessary x-rays for acute abdominal pain. One expert observed that this was resisted due to the risk that it may lead to several radiologists losing their jobs. Depending on the payment structure, generating revenue is sometimes not a direct factor for clinicians, but an indirect factor through the managers who want to maintain organizational financial health. Also, there exist risk that low-value care can increase when new care practices, especially new technologies, are reimbursed before the cost-effectiveness is evaluated. Two experts reported that Canada is more restrictive towards new technologies than the United States.

####  Industry

 According to the experts, the pharmaceutical and medical device industry has a powerful influence promoting the use of potentially unnecessary care. In addition to their direct contact with clinicians, they also exercise influence through education and guidelines. Experts shared that clinicians face advertising, which can lead them to believe that the product provides high-quality care. Product developers fund medical research and education, which can lead to biased knowledge. An expert mentioned a lawsuit that was initiated by the industry to encourage the use of opioids, and another expert mentioned the provision of a research fund as a reward for the use of their products. The industry can also influence political decisions to increase product sales. After it was announced that an orphan drug would not be reimbursed in the Netherlands for its high cost and lack of clinically relevant effect, the company that produced it put forward patient stories in the media, resulting in a re-evaluation and eventual reimbursement of the drug.

 Patients are also exposed to direct or indirect marketing. Whereas direct marketing of drugs is prohibited in Canada and the Netherlands, marketing the disease is legal. Companies raise awareness on for example prostate cancer and recommend the public to go to their doctor, increasing the necessary but also unnecessary use of their product. According to the experts, patient organizations sometimes receive financial support from the industry, which can help these organizations to support the patient population. It, however, also places them at risk of providing biased information to patients or the interests for which they advocate. For example, one expert described when a diabetes association argued for tighter hemoglobin a1c control, which would lead to more medicine being used.

####  Malpractice Litigation 

 Most experts agree that many clinicians are afraid of being sued by or getting complaints from patients and, therefore, practice defensive medicine and deliver more care. They described that a lawsuit is very upsetting personally and causes significant stress for clinicians. This fear can lead them to order more tests, procedures, or treatments that are unnecessary but provide additional documented evidence in support of their clinical decisions to prevent such lawsuits. Several Dutch experts suggested that malpractice lawsuits are less frequent in the Netherlands, possibly because the claims are lower, and therefore there might be less defensive medicine. According to the experts, it is not only the lawsuit but also the fear of making a mistake and having dissatisfied patients that motivate clinicians to overuse tests, procedures, or treatments.

###  Knowledge Factors

####  Evidence

 Several experts reported that the evidence for many tests, procedures, and treatments overestimates their effects in the real world. This bias is caused by publication bias, the ambition of researchers, and industry-sponsored research. An expert reported that the design of trials can be tainted by the wish to get favorable outcomes, making the evidence from these trials unreliable. In addition, it takes time for knowledge (biased or unbiased) to reach clinical practice. Clinicians need strong and solid evidence to accept that a care practice does not help the patient, when they have believed otherwise for years or when it makes sense that they work, based on pathophysiological reasoning. An expert stated that this biased evidence is not country-specific but affects the whole world.

####  Medical Education 

 Several experts said that, traditionally, medical education has been about thoroughness, which is now embedded in clinical practice patterns. Students are rewarded for being thorough but not for stewardship. This leads to the ‘more is better’ culture. Even practicing clinicians face potentially biased continued medical training that is sponsored by industry. Some experts also shared that clinicians work autonomously and rarely receive feedback so there is a lack of accountability mechanisms, although two experts reported that the United States has well-organized feedback systems in place, for example for antibiotic prescriptions. Experts expressed that more independent education and individual performance feedback could be vital tools to change clinician behavior.

###  Social Factors

####  Public Culture

 According to the experts, public culture is a significant factor promoting low-value care. Some individuals in the public hold assumptions, perceptions, and values in which more care and new technology is better, which lies in conflict with low-value care reduction efforts. This culture can be attributed to the quality of the information that is available to the public. This information includes overestimated benefits of treatment, underestimated harms, medicalized symptoms, anecdotal stories of missed diagnoses, and potentially biased industry-sponsored advertising. According to several experts, the society is less willing to accept risks or uncertainty. Several experts believed that this culture is a worldwide phenomenon. As a result of this, some patients request care from their clinician. It can be hard to reassure patients and explain to them that more care is not always better. Not all clinicians have the skills to have this conversation in a time-efficient way. An expert from the United States reported that low-value care is harder for United States citizens to understand, because there is also a lot of underuse and accessibility problems.

 Some experts argue that this factor is overestimated because many low-value care practices are not requested, such as routine lab tests for hospitalized patients. They also suggest that clinicians often misinterpret patients’ expectations and assume that they want care without asking them. Clinicians may be unconsciously driving the decision more than is sometimes assumed. Two Dutch experts reported that people in the Netherlands do not want care if it is not necessary. They suggest that this is attributable to their Calvinistic nature and attitude that pain is part of life.

####  Medical Culture

 Similar to the public, experts discussed that clinicians overestimate the benefits of treatments, underestimate the harms, and are influenced by anecdotal stories about rare diseases. The industry, fear of litigation, medical education and biased evidence contribute to this culture. Many clinicians are hooked on new technology and have the tendency to be ‘better safe than sorry’ to avoid uncertainty. An expert reported that not doing anything can feel counter-intuitive. Clinicians, also, desire to provide high-quality care and a positive experience for patients, which can guide them to meet patients’ wishes. Without the time for further conversation about care options, this can lead to decision-making supporting low-value care. The clinicians’ roles can be conflicting: they are expected to show compassion and support and to do what is in the patients’ best interest. An expert from Canada reported that medical centers in the United States and clinicians in private practice compete with each other to attract patients. They, therefore try to meet their wishes to obtain additionally requested labs and imaging, whereas in Canada this pressure from competition is less common. A Dutch expert agreed with this and stated that clinicians in the Netherlands are more used to withhold care from patients.

## Discussion

 Our study identified key factors that promote low-value care: a fee for service payment system, the pharmaceutical and medical device industry, the fear of being sued, the biased knowledge on care, medical education in which clinicians are trained to act, and the ‘more is better’ culture in the general public and in clinicians. The experts suggested that these factors have a synergistic relationship and that especially the industry strengthens the other factors. These factors are seen as the most important in all three countries, although the experts report several diffences in their payment structure, industry and malpractice policy, and culture regarding low-value care.

 Our study highlights the most important national level factors from the wide range that was identified by Saini et al.^16^ Whereas they conclude that the available resources, social and political contract, state of scientific knowledge, configuration of the system, and financing mechanisms influence the provision of care, the experts that we interviewed put more emphasis on the ‘more is better’ culture and fear of malpractice litigation. In addition, our analysis resulted in a different categorization than Saini et al. This could be explained by the focus of our study on overuse of low-value care and on the national level, as compared to Saini and colleagues focus on both overuse and underuse on all levels. Also, our study assessed experiences of experts in the field, whereas Saini et al drew their findings from literature. Several studies have identified barriers to reducing low-value care experienced by clinicians, such as patient expectations, efficiency, other doctors, malpractice fears, clinical uncertainty, lack of time, fear of bad outcomes and difficulty assessing medical records.^19,22-25^ Several of these barriers are reflected in the national-level factors that this study identified.

###  Implications for Research and Practice

 These seven factors can impact clinicians’ practices and are vital to consider when reducing low-value care. Choosing Wisely appeals to clinicians’ values and motivation to provide high-quality care, but it is implemented in a system and culture that impedes this. Therefore, it is crucial that we target these factors to enable the successful reduction of low-value care practices. Although creating this change can be challenging and requires policy and system changes, it potentially has a large, long-term impact on the provision of low-value care and the sustainability of our healthcare systems. Table 2 suggests several policy-related strategies per key factor. Below, several policies are discussed.

**Table 2:** Examples of Promising Policy-Related Strategies Per Factor

**Category**	**Factor **	**Examples Of Promising Policy-Related Strategies **
System factors	Payment structure	Moving from pay for performance toward other payment structures, such as capitation or value-based payment No longer reimbursing low-value care Fixed income for physicians
	Industry	Restricting industry ties in research and education
	Malpractice litigation	Reducing malpractice fear by protecting clinicians from the burden of a complaint
Knowledge factors	Evidence	Stimulating transparency on industry ties and independent research
	Medical education	Improving education on the harms of care Rewarding stewardship Providing individual performance feedback on low-value care
Social factors	Public culture	Information campaigns on low-value care Supporting clinicians to educate their patients
	Medical culture	Increasing awareness on culture and psychological preconceptions that drive low-value care

 With policy adjustments, healthcare systems are better supported to reduce low-value care by addressing these factors.^17,26-28^ For example, moving from pay for performance toward other payment structures, such as capitation or paying for quality instead of quantity can remove the pressure on clinicians to generate volumes.^29^ Most physicians in the United States and Canada receive a fee for service, while in the Netherlands, half of the specialists is salaried and general practitioners receive a capitation fee per registered patient. The United States is trying to shift towards value-based payment.^30^ The predominantly capitated National Health System in England,^31^ and no longer reimbursing care in Canada^13^ have shown to reduce low-value care use. In addition, local strategies such as global budgets for hospitals,^32^ a fixed budget contract between hospital and insurer and fixed income for specialists,^33^ and a cost accounting and shared savings program^34^ have potential to reduce low-value care.

 The influence of the pharmaceutical and medical device industry could be further restricted so patients and clinicians can base their decisions on unbiased and independent information. The United States and New Zealand are two of the few countries that still allow direct to consumer advertising. Regarding the marketing to clinicians, the United States already improved the transparency of payments with the Physician Payments Sunshine Act in 2010, although it has yet to be shown that disclosure affects marketing practices or the opinion of consumers.^35,36^ Other opportunities lie in restricting industry ties in research and education.^37^ It is important to note that, while the industry is considered to be an important promotor of low-value care, it also does a lot of good things to reduce underuse and improve the quality of care.

 Studies confirm that malpractice concerns are a reason to provide low-value care.^22,24,25,38^ As the experts in this study suggested, the Netherlands has a high claim rejection rate and relatively low payments compared to other countries.^39^ Nevertheless, Dutch physicians still experience fear of complaints.^40^ Also, although the number of lawsuits in the United States has been decreasing in the past 20 years, the practice of defensive medicine has continued.^41^ It is suggested that defensive medicine is self-reinforcing and research on how to break this mindset is necessary.^41^

 Several other researchers also recognize that medical and public culture promote low-value care.^27,42,43^ Unfortunately, this national or maybe even global culture is hard to recognize and change.^42^ On an organizational level, the High-Value Care Culture Survey can help to identify areas for improvement within the local culture.^44^ This survey has shown that training environment and reimbursement models are associated with high-value care culture.^45,46^ The lack of good evidence and our trust in the pathophysiological mechanism was also recognized as a reason for the use of treatments that lack benefit for the patient.^47^ Ubel and Asch suggested that awareness of the psychological preconceptions that drive low-value care can help clinicians to resist them.^48^ Regarding the public, their awareness of and responses to low-value care could be improved through the media.^49^ A review suggests that engaging patients within the patient-clinician interaction helps to reduce low-value care.^50^

 With this paper, policy-makers can gain an understanding of the key factors that lead to low-value care, which can help them to select solutions. As the antibiotic case in Box 1 illustrates, since there is not one factor that leads to low-value care alone, there is no single solution to address it. Depending on the magnitude of the factors and the country’s health system, further research can be undertaken and policy interventions can be considered. Quantifying the importance of the factors in each country would enable further research into country differences.

**Box 1. The Case of Inappropriate Antibiotic Use**

**Case: Antibiotics**

Antibiotic is often targeted in studies that focus on reducing low-value care.^8^ Inappropriate antibiotic use can cause adverse effects, wastes resources, and encourages antimicrobial resistance. Cognitive biases, pressure from patients, and lack of time promote antibiotic use.^51^

Interestingly, there is a considerable difference in the levels of antibiotic prescriptions between countries.^52^ This can be caused by several dimensions of culture,^53^ such as the way people deal with authority and uncertainty,^54^ promotional efforts of pharmaceutical companies, and reimbursement policies.^52,55^

Several policies have increased antibiotic stewardship. In 1997 Belgium limited the reimbursement of antimicrobial prophylaxis, which led to a sustained reduction,^56^ and these results were also found in Denmark.^57^ Also, restrictions on the marketing of pharmaceutical companies,^58^ and an increase in the number of general practitioners^59^ were related with less antibiotic prescriptions.

This case shows that for one low-value care practice there can be many factors that explain the variation between countries. Improving appropriateness of care is possible and understanding these factors within a specific country can help to develop successful interventions.

###  Strengths and Limitations

 A strength of our study is that through the Choosing Wisely network, we had the opportunity to interview experts with extensive experience with low-value care and de-implementation. A limitation of our study is that we did not quantify the importance of the factors identified, but this is an opportunity for further evaluation especially through country comparisons. Secondly, the factors that the experts described could be observed by them in practice, but since most of them keep up with medical literature, their responses could partly be a reflection of the literature. Thirdly, the experts mainly referred to low-value care delivered by physicians. This study cannot estimate whether low-value care in other disciplines, such as nursing or paramedics, is due to other factors. Fourthly, our convenience sample of experts might not be representative of experts more broadly. Also, 17 of the 18 experts were known by at least one of the authors before being approached for an interview. This previous relationship could have influenced their responses. Lastly, our results are based on experiences in three high-income countries. The presence and magnitude of factors differ between countries and healthcare systems. We, therefore, might have missed themes relevant to other, especially low- and middle-income countries.

## Conclusion

 The key factors promoting low-value care on a national level are the fee-for-service system, the pharmaceutical and medical device industry, fear of malpractice litigation, biased evidence and knowledge, medical education and the ‘more is better’ culture. These factors are seen as the most important in the United States, Canada and the Netherlands, although there are several differences in their payment structure, industry, and malpractice policy. Policy-makers and researchers that aim to reduce low-value care have experienced that clinicians are motivated to provide high-quality care for their patients, but they act in a system and culture that impedes this. Better awareness and understanding of these factors, and how other countries approach them can help clinicians to resist them and policy-makers to better support clinicians and medical centers to deliver high-value care to their patients.

## Acknowledgements

 We thank the experts that participated for their contribution to our study.

## Ethical issues

 The research protocol was sent to the Research Ethics Committee of the Radboud University Nijmegen medical center. The committee judged that ethical approval was not required under Dutch National Law (study number: 2017-3627).

## Competing interests

 Authors declare that they have no competing interests.

## Authors’ contributions

 EWV performed the interviews, led the analysis and drafted the manuscript. SAvD, RG, KB, GPW, and RBK contributed expertise in low-value care and healthcare overuse, and were involved in manuscript development and revisions. All authors read and approved the final manuscript.

## Funding

 This work was supported by ZonMw, a Dutch Organization for Health Research and Development (grant number 839201 002).

## Supplementary files

Supplementary file 1. Research Checklist.

Supplementary file 2. Interview Guide.

Supplementary file 3. Characteristics of the Experts That Participated.
