# Supplementary material for: Key Factors that Promote Low-Value Care: Views of Experts From the United States, Canada, and the Netherlands
Source: Int J Health Policy Manag. 2021 Jun 19;11(8):1514–21. doi: 10.34172/ijhpm.2021.53 (PMC9808325; doi:10.34172/ijhpm.2021.53)
Supplement: Supplementary file 1 — Research Checklist. [file ijhpm-11-1514-s001.pdf]

Article title: Key Factors that Promote Low-Value Care: Views of Experts From the United States, Canada, and The Netherlands

Journal name: International Journal of Health Policy and Management (IJHPM)

Authors' information: Eva W. Verkerk[1]*, Simone A. Van Dulmen[1], Karen Born[2], Reshma Gupta[3], Gert P. Westert[1], Rudolf B. Kool[1]

[1]Department of IQ Healthcare, Radboud Institute for Health Sciences, Radboud University Medical Center, Nijmegen, The Netherlands.

[2]Institute for Health Policy, Management & Evaluation, University of Toronto. Toronto, ON, Canada.

[3]University of California Health, Sacramento, CA, USA.

(*Corresponding author: Eva.verkerk@radboudumc.nl)

Supplementary file 1. Research Checklist

Research Checklist: the COREQ (Consolidated criteria for reporting qualitative research)

Supplement to: EW Verkerk, SA van Dulmen, K Born, R Gupta, GP Westert, RB Kool. Key Factors that Promote Low-Value Care: Views of Experts from the United States, Canada, and the Netherlands.

**Domain 1: research team and reflexivity**	
**Personal characteristics**	
**1. Interviewer**	EWV performed the interviews
**2. Credentials**	MScBS
**3. Occupation**	PhD candidate
**4. Gender**	Female
**5. Experience and training**	EWV is trained in qualitative research and interviewing and had experience interviewing healthcare professionals and policymakers.
**Relationship with participants**	
**6. Relationship established**	EWV met two participants earlier before interviewing them for this study, but she did not have a working or other relationship with them. She had not met the other participants before their interviews. Regarding the other authors that did not perform the interviews: 17 of the 18 participants were known by at least one of the other authors. One participant was not known personally by any of the authors before this study.
**7. Participant knowledge of the interviewer**	The reasons for the study were described in the e-mail with which they were approached and in the consent form. Participants were aware that EWV was a PhD candidate.

**8. Interviewer characteristics**	EWV discussed with some participants that she is not a clinician.
**Domain 2: study design**	
**Theoretical framework**	
**9. Methodological orientation and theory**	We used an inductive thematic analysis, in which the analysis is data-driven to guide researchers to create overarching themes based on coding without a pre-existing frame.
**Participant selection**	
**10. Sampling**	We selected from our professional networks a convenience sample of 20 policymakers, researchers, and other stakeholders with experience in identifying and reducing low-value care, distributed over the three countries. This was defined as having led at least one initiative to reduce low-value care, having evaluated such initiatives, or being responsible for reducing low-value care in an organization. We used purposive sampling to include experts from different institutes and programs and with different experiences with low-value care. For example, we selected experts involved in the Choosing Wisely campaigns, researchers that focus on low-value care, and leaders of various organizations that aimed to reduce low-value care. At the end of an interview, participants were asked if they could refer us to other experts. Five other experts were suggested, of which we approached two for an interview.
**11. Method of approach**	All experts were invited to participate and received information about the interviews by email.
**12. Sample size**	We interviewed 18 experts.
**13. Non-participation**	2 experts that we approached declined to participate, one because of pregnancy leave and one because of a change in position.
**Setting**	
**14. Setting of data collection**	We conducted face-to-face interviews with five Dutch experts and three Canadian experts at the location of their choice. Ten other interviews were by telephone, because of convenience.
**15. Presence of non-participants**	Only the experts and interviewer were present.
**16. Description of sample**	The sample was a mix of organizational leaders or policy makers, low-value care researchers or project leaders, or both. 61% of the experts had a background as a healthcare professional.
**Data collection**	
**17. Interview guide**	We used a semi-structured interview guide that was developed by all authors using existing literature on factors that promote low-value care. The interviewer tested the guide by interviewing a project manager from Choosing Wisely Canada. We added additional

	factors that emerged during the interviews in subsequent interviews. The final interview guide can be found in supplement 1.
**18. Repeat interviews**	We did not perform repeat interviews.
**19. Audio/visual recording**	The interviews were audio-recorded and transcribed.
**20. Field notes**	Field notes were made during most interviews in order to discuss adding additional factors to the interview guide with the other authors.
**21. Duration**	The interviews ranged in length from 27 minutes to 1,5 hours.
**22. Data saturation**	After analyzing interview 17 and 18, we concluded that no new information emerged and saturation was reached.
**23. Transcripts returned**	One expert who requested this was sent his transcript and returned it with additional comments. The other transcripts were not checked by the participants.
**Domain 3: analysis and findings**	
**Data analysis**	
**24. Number of data coders**	Two authors (EWV and SAvD) independently coded three interviews and discussed their coding until they reached consensus. EWV coded subsequent interviews and discussed her analysis regularly with SAvD.
**25. Description of the coding tree**	The final key factors that promote low-value care were presented in figure 1.
**26. Derivation of themes**	Themes were derived from the data.
**27. Software**	We used Atlas.ti 8.0.34 for coding.
**28. Participant checking**	Participants were not asked to provide feedback on the findings.
**Reporting**	
**29. Quotations presented**	Each theme was illustrated with a quotation in Table 1. Each participant is identified by their country and a number.
**30. Data and findings consistent**	There was good consistency between data and findings, we used a lot of the wording used by experts to describe the results. The experts were fairly consistent in the key factors that they mentioned.
**31. Clarity of major themes**	The major themes were clearly presented in the results and in figure 1.
**32. Clarity of minor themes**	Of each major theme, several examples and country-specific details mentioned by the experts were presented.

Reference: Tong A, Sainsbury P, Craig J. Consolidated criteria for reporting qualitative research (COREQ): a 32-item checklist for interviews and focus groups. *Int J Qual Health Care* 2007; **19**, 349–357. doi: 10.1093/intqhc/mzm042
