# Supplementary material for: Key Factors that Promote Low-Value Care: Views of Experts From the United States, Canada, and the Netherlands
Source: Int J Health Policy Manag. 2021 Jun 19;11(8):1514–21. doi: 10.34172/ijhpm.2021.53 (PMC9808325; doi:10.34172/ijhpm.2021.53)
Supplement: Supplementary file 2 — Interview Guide. [file ijhpm-11-1514-s002.pdf]

Article title: Key Factors that Promote Low-Value Care: Views of Experts From the United States, Canada, and The Netherlands

Journal name: International Journal of Health Policy and Management (IJHPM)

Authors' information: Eva W. Verkerk[1]*, Simone A. Van Dulmen[1], Karen Born[2], Reshma Gupta[3], Gert P. Westert[1], Rudolf B. Kool[1]

[1]Department of IQ Healthcare, Radboud Institute for Health Sciences, Radboud University Medical Center, Nijmegen, The Netherlands.

[2]Institute for Health Policy, Management & Evaluation, University of Toronto. Toronto, ON, Canada.

[3]University of California Health, Sacramento, CA, USA.

(*Corresponding author: Eva.verkerk@radboudumc.nl)

Supplementary file 2. Interview guide

Supplement to: EW Verkerk, SA van Dulmen, K Born, R Gupta, GP Westert, RB Kool. Key Factors that Promote Low-Value Care: Views of Experts from the United States, Canada, and the Netherlands.

I. Introduction

1. Introduction
2. Confirm consent
3. Recap study aim and purpose
4. Establish terminology:
    a. low-value care= care that is proven of little or no value to the patient
    b. de-implementation= the process of reducing use of low-value care
    c. nationwide= factors that are present in the whole country, that apply to many low-value care practices

II. Participant's background

5. Current function and involvement in the de-implementation of low-value care

III. Factors

I will first ask an open ended question on barriers and facilitators that you have experienced, and then we will go through several themes.

6. Open question: What nationwide factors promote low-value care in the United States/Canada/the Netherlands in your experience?

7. Opinion of several themes and influence on low-value care

a. Accessibility of care for patients
b. The general practitioner/primary care physician
c. The healthcare payment model
d. Malpractice liability
e. Performance measures or other health care quality control systems
f. Political stability or instability
g. The availability of data and measuring low-value care
h. The approval of new technologies
i. The pharmaceutical and medical device industry
j. Patient and consumer organizations
k. Health care provider organizations
l. Training and education of clinicians
m. Advertising for patients and care providers
n. General beliefs and values of the public
o. Attitude of clinicians
p. Receptivity of change
q. Prioritization of de-implementation
r. Is there anything missing?

8. Most important factors for your country?

IV. End interview

9. Anything else?

10. Who else would be interesting to talk to?

11. Thanks!
