# Supplementary material for: Key Factors that Promote Low-Value Care: Views of Experts From the United States, Canada, and the Netherlands
Source: Int J Health Policy Manag. 2021 Jun 19;11(8):1514–21. doi: 10.34172/ijhpm.2021.53 (PMC9808325; doi:10.34172/ijhpm.2021.53)
Supplement: Supplementary file 3 — Characteristics of the Experts That Participated. [file ijhpm-11-1514-s003.pdf]

Article title: Key Factors that Promote Low-Value Care: Views of Experts From the United States, Canada, and The Netherlands

Journal name: International Journal of Health Policy and Management (IJHPM)

Authors' information: Eva W. Verkerk[1]*, Simone A. Van Dulmen[1], Karen Born[2], Reshma Gupta[3], Gert P. Westert[1], Rudolf B. Kool[1]

[1]Department of IQ Healthcare, Radboud Institute for Health Sciences, Radboud University Medical Center, Nijmegen, The Netherlands.

[2]Institute for Health Policy, Management & Evaluation, University of Toronto. Toronto, ON, Canada.

[3]University of California Health, Sacramento, CA, USA.

(*Corresponding author: Eva.verkerk@radboudumc.nl)

Supplementary file 3. Characteristics of the experts that participated

Supplement to: EW Verkerk, SA van Dulmen, K Born, R Gupta, GP Westert, RB Kool. Key Factors that Promote Low-Value Care: Views of Experts from the United States, Canada, and the Netherlands.

	**Country**	**Clinician**<br>(is or has been a health care professional)	**Organizational leader/policy maker**<br>(can make health care policy or leads an organization)*	**Low-value care researcher/project leader**<br>(has led a project to reduce low-value care or studied an aspect of low-value care)*
**1**	US		X	
**2**	US			X
**3**	US	X	X	
**4**	US	X	X	X
**5**	US	X		X
**6**	US	X		X
**7**	CAN	X	X	
**8**	CAN			X
**9**	CAN		X	
**10**	CAN	X	X	X
**11**	CAN	X		X
**12**	NL/US	X		X
**13**	NL		X	
**14**	NL		X	
**15**	NL	X	X	X
**16**	NL		X	X
**17**	NL	X	X	
**18**	NL	X	X	

*Characterized by the authors

Abbreviations: US, United States; CAN, Canada; NL, the Netherlands.
